# A QUBO Formulation of Minimum Multicut Problem Instances in Trees for D-Wave Quantum Annealers

**DOI:** 10.1038/s41598-019-53585-5

**Published:** 2019-11-20

**Authors:** William Cruz-Santos, Salvador E. Venegas-Andraca, Marco Lanzagorta

**Affiliations:** 1 CU-UAEM Valle de Chalco, Hermenegildo Galeana 3, Valle de Chalco, Estado de México 56615 Mexico; 2 Tecnologico de Monterrey, Escuela de Ingenieria y Ciencias, Ave. Eugenio Garza Sada 2501, Monterrey, NL 64849 Mexico; 3 US Naval Research Laboratory, 4555 Overlook Ave. SW, Washington, DC 20375 USA

**Keywords:** Mathematics and computing, Computer science, Information technology, Information theory and computation

## Abstract

Quantum annealing algorithms were introduced to solve combinatorial optimization problems by taking advantage of quantum fluctuations to escape local minima in complex energy landscapes typical of NP − hard problems. In this work, we propose using quantum annealing for the theory of cuts, a field of paramount importance in theoretical computer science. We have proposed a method to formulate the Minimum Multicut Problem into the QUBO representation, and the technical difficulties faced when embedding and submitting a problem to the quantum annealer processor. We show two constructions of the quadratic unconstrained binary optimization functions for the Minimum Multicut Problem and we review several tradeoffs between the two mappings and provide numerical scaling analysis results from several classical approaches. Furthermore, we discuss some of the expected challenges and tradeoffs in the implementation of our mapping in the current generation of D-Wave machines.

## Introduction

Quantum Annealing algorithms (QA) constitute a paradigm of quantum computation focused on solving combinatorial optimization problems^1–9^. QA are based on quantum effects in order to escape local minima of a cost function by the effect of tunneling through barriers separating local minima. An example of physical realizations of quantum annealing are the D-Wave quantum computers which have proved to be advantageous to solve instances of some NP − hard problems^10^.

To solve a problem using the D-Wave architecture, we must express it as a quadratic unconstrained Boolean optimization problem (QUBO) or an equivalent Ising function defined on logical variables. Then, we embed the logical problem in the physical architecture of the quantum annealer by mapping logical variables and qubits. The final step consists of performing an annealing process and obtaining the results. There are some limitations of the D-Wave hardware such as the maximum number of physical qubits available (which is 2000 qubits in its most recent computer, the D-Wave 2000Q system); this limitation imposes a restriction on the size of the logical problem that can be embedded into the hardware. Another limitation is the connectivity of the current architecture which is in the form of a Chimera graph as, in order to represent a logical variable, it is necessary to use a chain of physical qubits. Finally, numerical precision is another key feature in the mathematical definition and computation of Ising model parameters due to the analog nature of the device and the presence of additive noise.

In this article, we present a QUBO transformation of a family of graphs, to be later used on a QA approach, in order to solve the Minimum Multicut Problem (MCC problem) which is of paramount importance in theoretical computer science and other disciplines such as computer vision. The MMC problem is defined as follows: given a graph *G* = (*V*, *E*) with associated positive weights to the edges and a list of vertex pairs (*s*_*i*_, *t*_*i*_), 1 ≤ *i* ≤ *k*, a minimum weight set of edges separating each pair of vertices in the list must be found.

The MMC problem is an NP − hard problem. In this paper, we study a QUBO formulation of the MCC problem on a family of connected trees, being this variant also NP − hard. We show two QUBO constructions for the studied problem. The first one is based on an intuitive linear programming formulation used to obtain a function of degree equal to the largest unique path between each pair (*s*_*i*_, *t*_*i*_), 1 ≤ *i* ≤ *k* in a tree. The second one is based on the basic idea that in order to separate each path between terminals *s*_*i*_ and *t*_*i*_, it is necessary to remove at most *k* edges. This basic observation was used to define a QUBO function of degree at most *k* − 1. Also, a general penalty approach for the QUBO function based on Boolean circuits is shown.

## Results

### Quantum annealing, pseudo boolean functions and QUBO formulations

The D-Wave hardware is a physical realization of QA which solves instances of the classical Ising problem on a transverse field. The Ising model problem is NP − hard^11^ and it is defined as follows: given a set of weights (called *fields*) *h*_*i*_ and *J*_*ij*_ (called *couplers*), find an assignment to the set of Ising spin variables **s** = {*s*_*i*_: 1 ≤ *i* ≤ *N*}, with *s*_*i*_ ∈ {−1, +1}, so as to minimize the energy function

1 $$E({\bf{s}})=\sum _{1\le i\le N}\,{h}_{i}{s}_{i}+\sum _{1\le i < j\le N}\,{J}_{ij}{s}_{i}{s}_{j},$$

|*h*_*i*_| ≤ 2 and |*J*_*ij*_| ≤ 1. Finding an assignment $${{\bf{s}}}^{\ast }={\rm{\arg }}\,{\min }_{{\bf{s}}\in {\{-1,+1\}}^{N}}\,E({\bf{s}})$$ is equivalent to finding the ground state of the corresponding Ising classical Hamiltonian,

2 $${H}_{p}=\sum _{1\le i\le N}\,{h}_{i}{\sigma }_{i}^{z}+\sum _{1\le i < j\le N}\,{J}_{ij}{\sigma }_{i}^{z}{\sigma }_{j}^{z}$$

where ${\sigma }_{i}^{z}$ is the Pauli matrix *z* acting on the *i*th particle. One of the schemes to realize QA is through adiabatic quantum evolution from the ground state of an initial Hamiltonian to a ground state of a final Hamiltonian^5^. According to this scheme a time-dependent Hamiltonian takes the form

$$H(\tau )=A(\tau ){H}_{0}+B(\tau ){H}_{p}$$

where *τ* = *t*/*t*_*a*_ for 0 ≤ *t* ≤ *t*_*a*_, is *t*_*a*_ is the total annealing time, and the initial Hamiltonian ${H}_{0}=-\,{\sum }_{1\le i\le N}\,{\sigma }_{i}^{x}$ responsible for quantum tunneling among the localized classical states corresponding to the eigenstates of Hamiltonian *H*_*p*_. Functions *A*(*τ*) and *B*(*τ*) are defined so that, at time *τ* = 0, the influence of Hamiltonian *H*_0_ is predominant against *H*_*p*_. As time evolution goes from *τ* = 0 to *τ* = 1, the influence of Hamiltonian *H*_*p*_ increases while *H*_0_ fades away.

Consider the Hamiltonian $H(t)=\tilde{H}(t/{t}_{a})=\tilde{H}(\tau )$ such that 0 ≤ *τ* ≤ 1, and let us denote by |*l*; *τ*〉 the instantaneous eigenvector of $\tilde{H}(\tau )$ corresponding to the instantaneous eigenvalue *λ*_*l*_(*τ*). We find that

$$\tilde{H}(\tau )|l;\tau \rangle ={\lambda }_{l}(\tau )|l;\tau \rangle $$

with

$${\lambda }_{0}(\tau )\le {\lambda }_{1}(\tau )\le \cdots \le {\lambda }_{{2}^{N}-1}(\tau ).$$

The Adiabatic theorem asserts that for sufficiently large *t*_*a*_,

3 $$\mathop{\mathrm{lim}}\limits_{{t}_{a}\to \infty }|\langle l=0;\tau =1|\psi ({t}_{a})\rangle |=1$$

for some solution *ψ*(*t*) to the Schrödinger equation with Hamiltonian *H*(*t*). Consequently, state *ψ*(*t*_*a*_) will be very close to the ground state of Hamiltonian *H*_*p*_ with high probability. A sufficient condition for the algorithm running time needed to satisfy the Adiabatic theorem is

$${t}_{a}\gg \frac{{\varepsilon }_{\max }}{{g}_{\min }^{2}}$$

where

$${g}_{{\min }}=\mathop{{\rm{\min }}}\limits_{0\le \tau \le 1}({\lambda }_{1}(\tau )-{\lambda }_{0}(\tau ))\,{\rm{such}}\,{\rm{that}}\,{\lambda }_{1}(1)\ne {\lambda }_{0}(1)$$

and

$${\varepsilon }_{{\max }}=\mathop{{\rm{\max }}}\limits_{0\le \tau \le 1}|\langle l=1;\tau |\frac{d\tilde{H}}{d\tau }|l=0;\tau \rangle |$$

#### Pseudo-boolean maps

Let **x** = {*x*_*i*_: 1 ≤ *i* ≤ *N*} be a set of *N* Boolean variables. A *pseudo-Boolean function* is a map $f:{\mathrm{\{0,}\mathrm{1\}}}^{N}\to {\mathbb{R}}$ represented as multi-linear polynomials:

4 $$f({\bf{x}})=\sum _{S\subseteq \{1,\ldots ,N\}}\,c(S)\prod _{j\in S}\,{x}_{j},$$

where $$c(S)\in {\mathbb{R}}$$.

The *degree* of *f*, denoted as deg(*f*), is the cardinality of the largest subset *S* ⊆ {1, …, *N*} for which *c*(*S*) ≠ 0. Similarly, the *size* of *f* denoted as size(*f*) is the total number of variable occurrences in it, i.e., size(*f*) = Σ_*S*:*c*(*S*)≠0_|*S*|.

Of our particular interest are the *quadratic* pseudo-Boolean functions ${f}_{ue}:{\{0,1\}}^{N}\to {\mathbb{R}}$ with deg(*f*_*ue*_) ≤ 2 expressed by polynomials of the form:

5 $${f}_{ue}({\bf{x}})=\sum _{1\le i\le N}\,{u}_{i}{x}_{i}+\sum _{1\le i < j\le N}\,{e}_{ij}{x}_{i}{x}_{j}$$

where ${u}_{i},{e}_{ij}\in {\mathbb{R}}$. The quadratic unconstraint binary optimization problem (QUBO) thus consists in finding an assignment ${{\bf{x}}}^{\ast }={\rm{\arg }}\,{{\rm{\min }}}_{{\bf{x}}\in {\{0,1\}}^{N}}\,{f}_{ue}({\bf{x}})$. Also, the quadratic pseudo-Boolean map coincides with the Ising model via variable substitution *x*_*i*_ = (1 + *s*_*i*_)/2 for *i* = 1, …, *N*.

It is often the case that a problem can be formulated in terms of pseudo Boolean expressions of degree greater than two, which can subsequently be reduced to a QUBO function. However, this reduction commonly implies adding new variables to the former problem^12–14^ (an introduction to NP−hard problems and quantum annealing-based algorithms can be found in^15^).

In the following sections, we address the QUBO formulation of the MMC problem which is of utmost theoretical and practical importance in the computer science community.

### Quantum formulation of the MMC problem

An undirected weighted graph has the form *G* = (*V*, *E*, *w*) where *V* is a finite set of *vertices*, *E* ⊆ *V*^(2)^ = {{*u*, *v*}|*u*, *v* ∈ *V*} is a set of unordered pairs of vertices or *edges* and $w:E\to {{\mathbb{R}}}^{+}\cup \{0\}$ is a *weighted* map. Let us now define the MCC problem.

#### Problem 1

(**MMC problem**) *Given a weighted graph G* = (*V*, *E*, *w*) *and a list of vertices pairs* (*s*_*i*_, *t*_*i*_), 1 ≤ *i* ≤ *k*, *find a multicut with minimum weight*, *i*.*e*., *a subset E*′ ⊆ *E such that the removal of E*′ from E *disconnects s*_*i*_ *from t*_*i*_ *for every pair* (*s*_*i*_, *t*_*i*_), *where the weight of E*′ *is given as* ${\sum }_{\{u,v\}\in E^{\prime} }\,w(u,v)$.

For *k* = 1, the MMC problem reduces to the min-cut/max-flow problem that can be solved in polynomial time^16^. The problem is also tractable when *k* = 2, by using two applications of the min-cut/max-flow algorithm^17^. It becomes NP − hard when *k* ≥ 3 for general graphs, but can be solved in polynomial time for planar graphs for any fixed *k*^18^. It has been proved that the MMC problem is NP − hard and MAXSNP − hard on trees^18,19^. The MAXSNP − hardness of the MCC problem implies that no polynomial time approximation scheme exists unless P = NP.

The MMC problem can be applied in many areas such as telecommunication, routing, VLSI design and circuit partitioning^20,21^. In the following, we study the QUBO formulation of the MMC problem for the case when *G* is a tree.

#### A direct mapping of MMC into QUBO

Let *G* = (*V*, *E*, *w*) be a weighted graph and (*s*_*i*_, *t*_*i*_), 1 ≤ *i* ≤ *k* a list of vertex pairs. For each edge *e* ∈ *E*, we introduce a Boolean variable *x*_*e*_ such that *x*_*e*_ = 0(1), if *e* is (not) considered for a MMC in *G*. If graph *G* is restricted to be a tree, then there exists a unique path in *G* for every pair of vertices. We define the unique path from *s*_*i*_ to *t*_*i*_ in *G* as *p*_*i*_ where its *length l*_*i*_ is equal to the number of edges that it crosses. The *diameter* of a tree *T* = (*V*, *E*), denoted as *diam*(*T*), is equal to the maximum path length between every pair of vertices in *T*.

Let $P={\cup }_{i=1}^{k}\,{p}_{i}$ be the union of all edges in the paths *p*_*i*_ for *i* = 1, …, *k*. Let us to define the following function,

6 $$H={H}_{w}+{H}_{pen}$$

where

7 $${H}_{w}=\sum _{e\in P}\,w(e)(1-{x}_{e})$$

and

8 $${H}_{pen}={\lambda }_{P}\mathop{\sum }\limits_{i=1}^{k}\,\prod _{e\in {p}_{i}}\,{x}_{e}.$$

In (6), *H*_*w*_ expresses the weight of the selected edges to be removed and *H*_*pen*_ serves to add a penalty value when the considered edges do not correspond to a MMC. Based on this construction, the MMC problem on trees is equivalent to minimizing *H* over all possible assignments to the Boolean variables *x*_*e*_.

The penalty term satisfies 0 ≤ *H*_*pen*_ ≤ *kλ*_*P*_, where *λ*_*P*_ is a positive constant. In particular, when all paths are disconnected, *H*_*pen*_ = 0 which means that at least one edge was removed in every path *p*_*i*_ for *i* = 1, …, *k*. We must ensure that *λ*_*P*_ is big enough so that the weight of an invalid multicut is greater than the weight of any valid multicut. The value of *λ*_*P*_ can be upper bounded by ${\lambda }_{P}={\sum }_{e\in P}\,w(e)$. The degree of *H*_*pen*_ is equal to the maximum length of the paths *p*_*i*_ for *i* = 1, …, *k*. In other words,

$${\rm{\deg }}\,({H}_{pen})=\,{\rm{\max }}\,\{{l}_{i}|i=1,\ldots ,k\}.$$

In order to optimize (6) via QA, we need to write it in QUBO form (possibly by using section 1.1). For instance, assume that each path *p*_*i*_ does not have a trivial length, i.e. *l*_*i*_ > 2; using the reduction method in^14^, the penalty term *H*_*pen*_ can be transformed into a quadratic function adding a total of ${\sum }_{i=1}^{k}\,\lfloor \frac{{l}_{i}-1}{2}\rfloor$ new variables. This transformation is as follows:

- If *l*_*i*_ is even then
  9 $$\prod _{e\in {p}_{i}}\,{x}_{e}={S}_{2}+\mathop{{\rm{\min }}}\limits_{{\bf{y}}\in {\{0,1\}}^{{k}_{1}}}\{B-2A{S}_{1}\}$$
- If *l*_*i*_ is odd then we find that

10 $$\prod _{e\in {p}_{i}}\,{x}_{e}={S}_{2}+\mathop{{\rm{\min }}}\limits_{{\bf{y}}\in {\{0,1\}}^{{k}_{1}}}\{B-2A{S}_{1}+{y}_{{k}_{1}}({S}_{1}-{l}_{i}+1)\}$$

where

11 $${S}_{1}=\sum _{e\in {p}_{i}}{x}_{e},\,{S}_{2}=\sum _{e,e^{\prime} \in {p}_{i}e\ne e^{\prime} }\,{x}_{e}{x}_{e^{\prime} },\,A=\mathop{\sum }\limits_{j=1}^{{k}_{1}}\,{y}_{j},\,B=\mathop{\sum }\limits_{j=1}^{{k}_{1}}\,(4j-1){y}_{j},$$

and ${\bf{y}}=({y}_{1},\ldots ,{y}_{{k}_{1}})\in {\{0,1\}}^{{k}_{1}}$ is a vector of *k*_1_ new variables, and ${k}_{1}=\lfloor \frac{{l}_{i}-1}{2}\rfloor$.

The proposed QUBO function obtained from (6) using the Ishikawa reduction method presented in^14^ has as main advantage that its coefficients are small. This is a highly desirable property when programming the D-Wave quantum annealer because of its limited hardware precision to specify the values of Ising model parameters *h*_*i*_ and *J*_*ij*_.

A disadvantage of function *H* given in (6) is that its degree depends on the length of the paths. In subsection 2.2 we present another method to build a low degree *H*_*pen*_, based on the number of edges shared between paths.

#### Construction of *H*_*pen*_ based on crossing paths

Although several techniques exist for degree reduction of an arbitrary pseudo-Boolean function into a quadratic one, it is preferable to construct an initial expression of degree as low as possible. In this method, the key idea behind buliding a low degree function *H*_*pen*_ is to notice that a multicut consists of a subset of edges of cardinality of at most *k*. This condition imposes a restriction on the number of edges that could be removed in order to disconnect each path. Based on this observation, our goal will be to construct a new penalty function ${H}_{pen}^{^{\prime} }$ such that it will penalize all subsets of edges of cardinality larger than *k*.

For any *i*, *j* ∈ {1, …, *k*}, let *ζ*_*ij*_ be a characteristic function defined as

12 $${\zeta }_{ij}=\{\begin{array}{ll}1 & {\rm{if}}\,{p}_{i}\cap {p}_{j}\ne \varnothing ,\,i\ne j\\ 0 & {\rm{otherwise}}.\end{array}$$

We say that two paths *p*_*i*_ and *p*_*j*_ *intersect* if *ζ*_*ij*_ = 1. In particular, if *i* = *j* then *ζ*_*ij*_ = 0. For each *j* ∈ {1, …, *k*}, the quantity ${c}_{j}={\sum }_{i=1}^{k}\,{\zeta }_{ij}$ is equal to the number of paths *p*_*i*_ that intersect with path *p*_*j*_. Thus, the maximum number of paths than can intersect with a path *p*_*j*_ is *k* − 1. It is assumed that the number of intersections cannot be greater than the length of a given path.

For any *j* ∈ {1, …, *k*} and *η* ∈ {1, …, *k* − 1}, let *C*_*ηj*_ be given by

13 $${C}_{\eta j}={(\eta -{l}_{j}+\sum _{e\in {p}_{j}}{x}_{e})}^{2}$$

where *C*_*ηj*_ = 0 if the number of edges to be removed from path *p*_*j*_ equals *η*, and *C*_*ηj*_ > 0 otherwise. The new penalty term is

14 $${H}_{pen}^{^{\prime} }={\lambda }_{P}\mathop{\sum }\limits_{j=1}^{k}\,\mathop{\prod }\limits_{\eta =1}^{{c^{\prime} }_{j}}\,{C}_{\eta j}$$

where ${c^{\prime} }_{j}=1$ if *c*_*j*_ = 0, 1, and ${c^{\prime} }_{j}={c}_{j}$ if *c*_*j*_ > 1.

Function ${H^{\prime} }_{pen}$ has as objective to penalize sets of edges of cardinality greater than *k* rather than penalizing sets of edges that do not correspond to a multicut as the term *H*_*pen*_ does. ${H^{\prime} }_{pen}=0$ if and only if every path *p*_*j*_ is disconnected, and ${H^{\prime} }_{pen} > 0$ otherwise. The degree of ${H^{\prime} }_{pen}$ is at most twice the maximum number of intersections in all paths, i.e.

$${\rm{\deg }}\,({H^{\prime} }_{pen})\le 2\,{\rm{\max }}\,\{{c^{\prime} }_{j}|j=1,\ldots ,k\}\le 2(k-1)$$

which does not depend on the length of paths *p*_*i*_ in *G*.

The penalty function ${H^{\prime} }_{pen}$ can be reduced into a QUBO function using the method in^12^, producing a quadratic expression, the size of which is polynomially bounded in size(${H^{\prime} }_{pen}$) and the number of new variables is *O*(*n*^2log*d*^) where *d* = deg(${H^{\prime} }_{pen}$) and *n* = |*P*|. A disadvantage of this method is that the resulting quadratic function has many large coefficients and also introduces many positive quadratic terms. These two effects make the minimization of the resulting function a hard problem^22,23^.

#### A Boolean circuit construction for ${H^{\prime} }_{pen}$

Let **x** = {*x*_*i*_|1 ≤ *i* ≤ *N*} be a set of *N* Boolean variables, and let *b* be a positive integer. Based on section 2.2, the penalty term ${H^{\prime} }_{pen}$ can be written in terms of a pseudo Boolean function *ϕ*_*cj*_ as follows

15 $${H^{\prime} }_{pen}({\bf{x}})={\lambda }_{P}\mathop{\sum }\limits_{j=1}^{k}\,{\varphi }_{{c^{\prime} }_{j}}({\bf{x}})$$

satisfying that

16 $${\varphi }_{{c^{\prime} }_{j}}({\bf{x}})=\{\begin{array}{ll}0 & {\rm{if}}\,{l}_{j}-{c^{\prime} }_{j}\le \sum _{e\in {p}_{j}}\,{x}_{e}\le {l}_{j}-1,\\ \ge \,b & {\rm{otherwise}}.\end{array}$$

Function *ϕ*_*cj*_ can be constructed if we consider a Boolean circuit *g*: {0, 1}^*N*^ → {0, 1} such that *g*(**x**) = 1 when *ϕ*_*cj*_(**x**) > 0, and *g*(**x**) = 0 otherwise. This Boolean circuit can always be expressed as a pseudo Boolean function *ϕ*_*g*_ = ${\varphi }_{{c^{\prime} }_{j}}$ such that they both have the same values at every point. Let us formalize this result. A *disjunctive form* (DF) is an expression of the form

17 $$\phi =\underset{k=1}{\overset{m}{\vee }}(\mathop{\wedge }\limits_{i\in {A}_{k}}{x}_{i}\wedge \mathop{\wedge }\limits_{j\in {B}_{k}}\neg \,{x}_{j})$$

where $${A}_{k}\cap {B}_{k}=\rlap{/}{0}$$ for *k* = 1, …, *m*.

A DF is said to be *orthogonal* if $({A}_{k}\cap {B}_{l})\cup ({A}_{l}\cap {B}_{k})\ne \rlap{/}{0}$ for all *k*, *l* ∈ {1, …, *m*} with *k* ≠ *l*. An orthogonal DF *φ* represents a Boolean circuit *g* : {0, 1}^*N*^ → {0, 1} if the truth value points of *g* coincide with the truth value points of *φ*. Furthermore, every Boolean circuit *g* : {0, 1}^*N*^ → {0, 1} represented by an orthogonal DF has an associated multilinear polynomial introduced in^24^ and presented in (18),

18 $${\varphi }_{g}({\bf{x}})=\mathop{\sum }\limits_{k=1}^{m}(\prod _{i\in {A}_{k}}\,{x}_{i}\prod _{j\in {B}_{k}}\,(1-{x}_{j})).$$

The Boolean circuit *g*: {0, 1}^*N*^ → {0, 1} can be constructed using the standard Karnaugh map procedure^25^. This technique receives as input a truth table on *N* Boolean variables and returns a Boolean circuit expressed as a sum of products (SOP) of the involved variables which can be implemented using AND, OR and NOT gates. After obtaining the Boolean formulae in terms of the elementary Boolean gates, the NOT gate is interpreted as ¬*x* = 1 − *x*.

For instance, the corresponding pseudo-Boolean function obtained through Karnaugh maps as SOP can be expressed as

19 $${\varphi }_{{c^{\prime} }_{j}}({\bf{x}})=\mathop{\sum }\limits_{i=1}^{{c^{\prime} }_{j}}\,\sum _{A\subseteq {p}_{j},|A|={l}_{j}-i}\,\prod _{e\in A}\,(1-{x}_{e})+\prod _{e\in {p}_{j}}\,{x}_{e}.$$

As it can be seen in (19), the degree of function ${\varphi }_{{c^{\prime} }_{j}}$ is equal to the length of path *p*_*j*_. This penalty function has the same degree as the expression given in (8), however, the former has the property that penalizes multicuts of cardinality greater than *k*.

### Experiments and Discussion

**Figure 1:**
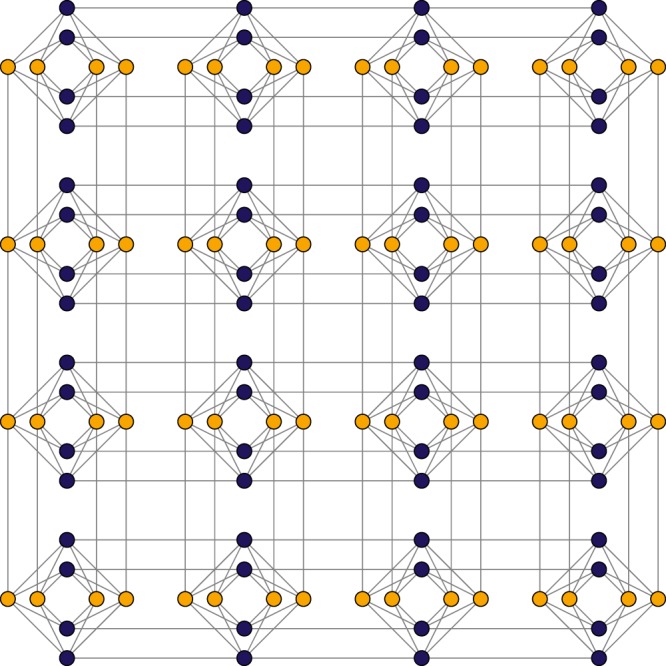
Chimera graph topology of 4 × 4 blocks.
  At this step, a key feature to remember is physical resources vs logical variables as, in order to embed a logical problem with arbitrary interconnectivity, we will frequently need a larger number of physical qubits than the number of logical variables; moreover, if the number of logical variables scales up rapidly, we may run out of physical qubits.
  The estima tion of the minim um number of physical qubits required to embed a logical problem is an active area of research^26,27^. The Chimera graph architecture implements an Ising model, hence the embedding process maps logical variables into Ising variables. Another important consideration is that the D-Wave processor has limited precision to represent Ising coefficients; consequently, the c oefficients of the Q UBO expression should not be too large so that they can be correctly mapped into the quantum pro cessor and results can be discriminated from noise level.
(iv) Finally, the annealing proc ess is initiated to find the minimum energy configuration in which the solutio n of the problem is codified.

We now present our algorithms and corresponding outcomes, as well as the limitations of our proposal. First, let us review the road map (steps) for solving an optimization problem using a quantum annealing approach:

(i) Select an optimization combinatorial problem (in our case, the MMC problem).
(ii) Construct a pseudo-Boolean function on binary variables for the selected optimization problem, so that those assignments that minimize the expression also correspond to solutions of the given problem. Since the D-Wave quantum processor has a specific architecture, it only supports pairwise interaction between qubits. Hence, the constructed pseudo-Boolean function must be of degree two, i.e., a QUBO expression. It is not always possible to directly obtain a QUBO expression for a given optimization problem, so in practice we have a high degree pseudo-Boolean function which is to be transformed into a QUBO expression at a later stage.
(iii) Once we have computed a QUBO expression for the selected problem, the next step is to embed the logical problem into the fixed architecture of the quantum processor. This architecture is represented as a Chimera graph which has a limited interconnectivity (see Fig. 1 for an example of a Chimera graph).

For steps (i) and (ii), we consider the QUBO formulations of the MMC pr oblem present ed in section 2: the formulatio n with direct reduction, the one base d on crossing of paths and the one based on logical circu its. For the last two cases, the reduction methods cited in Section 1.1 are use d to obtain the QUBO functions.

In the following section, we consider the mapping problem presented above in step (iii), namely mapping logical variables in a QUBO function to physical qubits in the actual hardware architecture.

#### Minor embedding

The D-Wave processor can be represented by an architecture known as a *Chimera graph* which consists of an *M* × *N*-lattice of blo cks each one having 2*L* physical qubits for a total of 2*MNL* qubits (see Fig. 1). Each block in the Chimera graph is a *L*-bipartite graph and each physical qubit is connected with at most six other qubits. To solve a problem using a D-Wave processor, it is necessary to represent an Ising/QUBO problem as a subgraph of the Chimera. However, it is seldom possible to find a one-to-one mapp ing of logical variables with physical qubits.

The method to find an equivalent subgraph into the Chimera to a given logical problem expressed as an Ising/QUBO function is called *minor embeddi ng* and it is stated as follows:

##### **Problem 2**:

(**Minor embedding**) *Given a Chimera graph* ${{\mathscr{G}}}_{M,N,L}$ *and a logical graph G* = (*V*, *E*), *find a subgraph in* ${{\mathscr{G}}}_{M,N,L}$ *such that*

1. Each vertex *j* ∈ *V* is mapped to a connected subtree *T*_*j*_ in ${{\mathscr{G}}}_{M,N,L}$.
2. Each edge {*i*, *j*} ∈ *E* must be mapped to at least one coupler in ${{\mathscr{G}}}_{M,N,L}$.

Finding a minor embedding which uses the minimum number of qubits is a hard problem in general^26^. However, there are heuristic algorithms that obtain approximated minor embeddings in polynomial time. One of these techniques is the one proposed in^28^ which maps a logical variable to a chain of qubits. The main disadvantage of these techniques is that they create long chains of qubits to allow the connectivity of the logical problem into the Chimera.

In this paper, we use the D-Wave solver application programming interface (SAPI) which provides heuristic algorithms for minor embedding. Heuristic algorithms are constrained by a peculiar property: their time complexity (i.e., their worst and average runtime) is usually lower than exhaustive search algorithms at the cost of producing approximative solutions most of the time and optimal results only rarely. In our case, SAPI heuristic algorithms produce different embeddings that are not always optimal with respect to the number of physical qubits. For each or our experiments, we have run the heuristic embedding algorithms twenty times and have chosen only those embeddings that have shorter qubit chains. This process is carried out on a conventional digital computer, before the adiabatic process.

#### Instance problem generation

For this paper, we have generated random instances of the MMC problem, namely random trees, according to the following procedure: (1) generate a connected random graph^16^, (2) compute their corresponding spanning tree (ST) and (3) assign integer positive weights to the edges of the obtained ST. Here, we consider the generation of connected random graphs using the Erdös-Rényi (ER) and the Watts-Strogatz (WS) models. In the ER model, a random graph *G*(*n*, *p*) with *n* vertices is constructed by including each possible edge with a probability *p* independently from every other edge. In the WS model, a random graph *G*(*n*, *r*, *β*) with *n* vertices is constructed by creating an initial ring of vertices with each vertex connected to its *r* nearest neighbors. Thus, replace every possible edge {*u*, *v*} with a new edge {*u*, *v*′} with probability *β*, duplicated edges are forbidden, but original edges may end up being reinstated.

**Figure 2:**
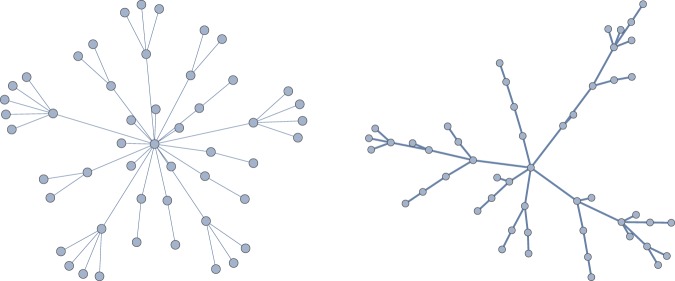
Example random trees: (left) using the Erdös-Rényi model for *n* = 50 and *p* = 0.3, and (right) using the Watts-Strogatz model with *n* = 50, *r* = 2 and *β* = 0.12.

Figure 2 shows some examples of trees generated using the ER and WS models. We chose the ER and WS models because they allow us to generate trees with small and large diamete rs, respectively. For instance, Fig. 2 (left) shows a tree with a diameter of 4 using the ER model and Fig. 2 (right) shows a tree with a diameter of 10 using the WS model, in both cases the number of vertices in the random graph are the same.

**Figure 3:**
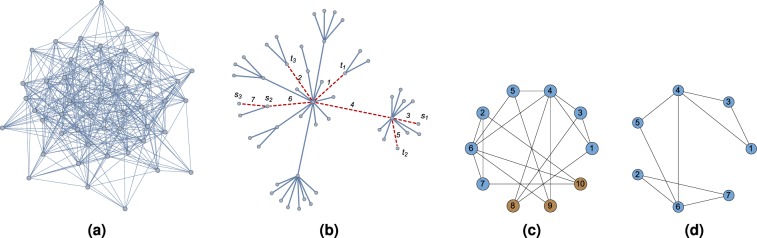
(**a**) ER random graph *G*(50, 0.3), (**b**) corresponding spanning tree, (**c**) logical graph of the QUBO reduction of functions (20) and (22), and (**d**) logical graph of the QUBO expression given in (21).

Let us consider an example of how the QUBO formulations given in Section 2 for the MMC problem are constructed. Figure 3(a) shows a random ER graph for 50 vertices and probability *p* = 0.3, and Fig. 3(b) shows its corresponding random spanning tree. Assume an instance of the MMC problem with unitary weights for *k* = 3 and set of pairs {(*s*_1_, *t*_1_), (*s*_2_, *t*_2_), (*s*_3_, *t*_3_)}; from Fig. 3(b), it can be seen that paths *p*_*j*_, *j* = 1, 2, 3 contain edges {1, 3, 4}, {4, 5, 6} and {2, 6, 7}, respectively. Notice that the length of paths *p*_*j*_ is *l*_*j*_ = 3 for *j* = 1, 2, 3, and paths *p*_1_ and *p*_2_ share the edge 4, and paths *p*_2_ and *p*_3_ share the edge 6.

The direct mapping given in (6) for the instance of the MMC problem in Fig. 3(b) is

20 $$H=7-{x}_{1}-{x}_{2}-{x}_{3}-{x}_{4}-{x}_{5}-{x}_{6}-{x}_{7}+7{x}_{3}{x}_{4}{x}_{1}+7{x}_{4}{x}_{5}{x}_{6}+7{x}_{2}{x}_{6}{x}_{7}.$$

The logical graph obtained from the QUBO reduction of (20) can be seen in Fig. 3(c) where the blue color vertices are the original problem variables and the brown color vertices are the introduced new variables. Notice that the induced subgraphs from vertices {1, 3, 4} and {4, 5, 6} share vertex 4, and the induced subgraphs from vertices {4, 5, 6} and {2, 6, 7} share vertex 6. Also, notice that vertices {1, 3, 4} together with the new variable 8 form a complete graph; the same is also true for vertices {4, 5, 6} and {2, 6, 7} together for new variables 9 and 10, respectively. From the above, it can be concluded that if the paths do not intersect each other, then their corresponding logical graphs will be disconnected.

On the other hand, the mapping based on crossing paths given in (14) for the instance of the MMC problem in Fig. 3(b) is

21 $$\begin{array}{rcl}H & = & 91-22{x}_{1}-22{x}_{2}-22{x}_{3}-43{x}_{4}-22{x}_{5}-43{x}_{6}-22{x}_{7}+14{x}_{3}{x}_{1}+14{x}_{4}{x}_{1}\\  &  & +\,14{x}_{3}{x}_{4}+14{x}_{4}{x}_{5}+14{x}_{2}{x}_{6}+14{x}_{4}{x}_{6}+14{x}_{5}{x}_{6}+14{x}_{2}{x}_{7}+14{x}_{6}{x}_{7}\end{array}$$

which is already in QUBO form since we penalize cuts of cardinality at most three. In other words, each path *p*_*j*_, *j* = 1, 2, 3 will be disconnected by removing at most one edge. Figure 3(d) shows the logical graph of expression (21) where no new variables are used. Notice that only the induced subgraphs of vertices {1, 3, 4}, {4, 5, 6} and {2, 6, 7} are shown.

Finally, the Karnaugh mapping given in (19) for the instance of the MMC problem in Fig. 3(b) is

22 $$\begin{array}{rcl}H & = & 70-15{x}_{1}-15{x}_{2}-15{x}_{3}-29{x}_{4}-15{x}_{5}-29{x}_{6}-15{x}_{7}+7{x}_{3}{x}_{1}\\  &  & +\,7{x}_{4}{x}_{1}+7{x}_{3}{x}_{4}+7{x}_{4}{x}_{5}+7{x}_{2}{x}_{6}+7{x}_{4}{x}_{6}+7{x}_{5}{x}_{6}+7{x}_{2}{x}_{7}\\  &  & +\,7{x}_{6}{x}_{7}+7{x}_{3}{x}_{4}{x}_{1}+7{x}_{4}{x}_{5}{x}_{6}+7{x}_{2}{x}_{6}{x}_{7}\end{array}$$

whose logical graph coincides with the QUBO reduction of (20).

#### Problem scaling

**Figure 4:**
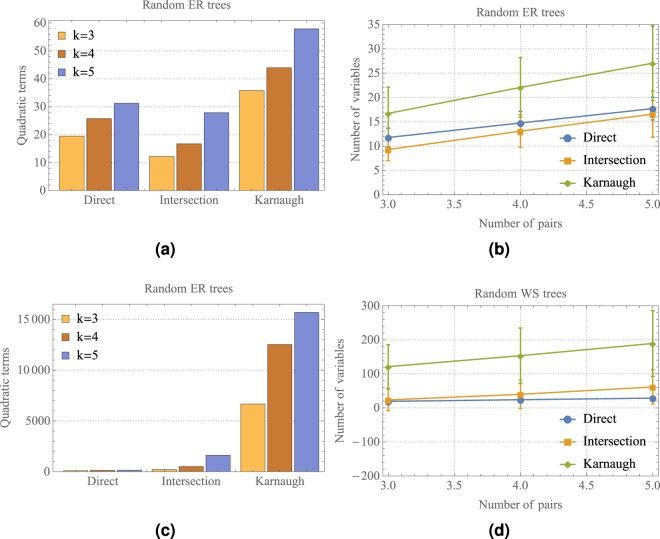
Comparison of the number of quadratic terms generated using the direct, intersection and Karnaugh methods.

Figure 4 shows a comparison of the number of quadratic terms generated by the proposed QUBO formulations presented in sections 2.1, 2.2 and 2.3, that will be named as the direct, intersection and Karnaugh methods, respectively. We generated random trees using the ER and WS models with probabilities *p* = 0.3 and *β* = 0.12, for *n* = 50 and number of pairs *k* = 3, 4, 5. We generate random WS trees with paths between the pairs (*s*_*i*_, *t*_*i*_) of length less than 8. For each random tree model used, the corresponding QUBO function was constructed using the direct, intersection or Karnaugh methods. The energy function given in (6) was used for the three methods with different penalty terms. The direct method corresponds to the penalty term as presented in (8), the intersection method has as penalty term the expression shown in (14), and the Karnaugh method has as penalty term the expression given in (19). We use the reduction method of Ishikawa^14^ explained in section 2.1 as well as Freedman’s method^13^, to obtain the corresponding QUBO function.

Figure 4(a) presents is the average number of quadratic terms over 100 ER random trees, generated using the direct, intersection and Karnaugh methods for *k* = 3, 4, 5. Figure 4(b) shows their corresponding average number of variables after the degree reduction of the constructed QUBO functions shown in Fig. 4(a). The vertical lines in Fig. 4(b,d) indicate a standard deviation. As it can be seen in the Fig. 4(a,b), the intersection method uses the minimum number of variables to represent the MMC problem and the Karnaugh method requires more variables to represent the same problem. It is important to mention that the average diameter of the generated ER random trees was 4. We choose *n* = 50 since the number of quadratic terms is an increasing function with respect to *n*. Similarly, Fig. 4(c,d) show the average number of quadratic terms using WS random trees for the direct, intersection and Karnaugh methods. It is remarkable that Karnaugh’s method requires a huge number of quadratic terms and variables in comparison with the direct and intersection methods. On the other hand, the direct method uses the minimum number of quadratic terms. In this case, the average diameter of the generated WS random trees was 10.

**Figure 5:**
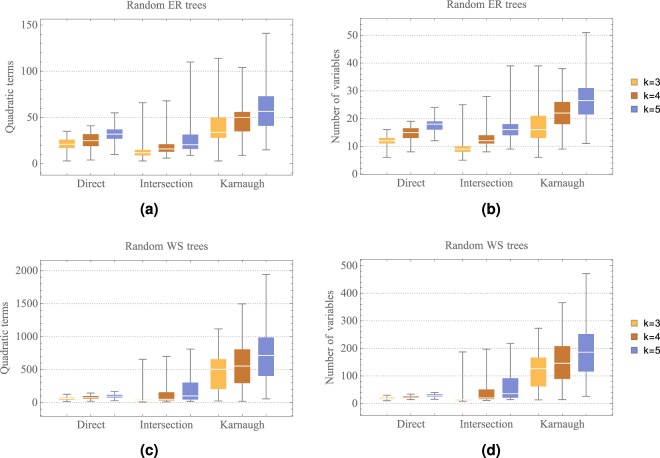
Descriptive comparison of the number of quadratic terms and number of variables generated using the direct, intersection and Karnaugh methods.

Figure 5 presents an even more descriptive comparison of the number of quadratic terms and number of variables obtained after degree reduction, for the direct, intersection and Karnaugh methods. The area in each box represents the interquartile range, the statistical median as a horizontal line in the box, and its vertical lines the lower and upper whiskers. The data shown in Fig. 5(a–d) correspond to the same data shown in Fig. 4. As can be noted from Fig. 5, the lower and upper whiskers for the Karnaugh method are distant from their median, which explains the standard deviation in Fig. 4(b,d).

In view of these scaling results, it can be seen that Karnaugh’s construction given in (19) has terms of degree equal to the length of the paths between pairs (*s*_*i*_, *t*_*i*_). Thus, when applying a reduction method on (19), the number of quadratic terms increases, as opposed to the method given in (14) whose degree depends on the number of crossing paths. As a consequence, the dimension of the search space of its corresponding optimization problems also increases.

#### Classical solution performance

In order to test the performance of obtaining solutions using the proposed formulations, the qbsolv tool was used to solve large QUBO problems by partitioning into subproblems targeted for execution on a D-Wave system^29^. The qbsolv tool returns approximated solutions of large QUBO problems. The experiments were performed on a Desktop computer MacBook Air with a Intel Core i5 processor at 1.3 GHz and 4 GB of RAM. We generated 80 random trees for *n* = 50 and *k* = 3 using the ER and WS models, and for each random tree, their corresponding QUBO functions were constructed using the direct, intersection and Karnaugh methods.

Before mapping a QUBO expression to Ising, constant terms are omitted without changing the original problem because constant terms cannot be represented in the graph topology. For the D-Wave SAPI software, an auto-scaling mode is used which utilizes the largest possible parameter ranges. In the case of the qbsolv tool we do not have precision limitations on the parameters, so each QUBO instance is directly submitted.

**Figure 6:**
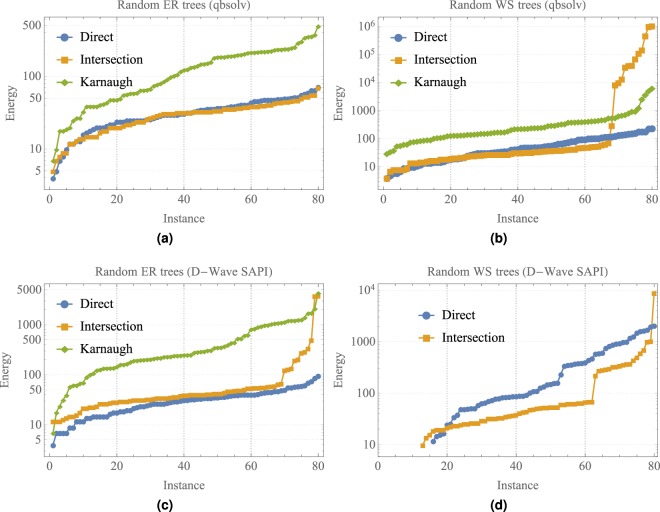
Sorted energy solutions obtained using the qbsolv and D-Wave simulator over 80 random instances using the direct, intersection and Karnaugh QUBO constructions.

Figure 6(a,b) show the energy solutions obtained using qbsolv for ER and WS random trees, respectively. To compare the approximated energies solutions, they were sorted in an increasing order over the same set of instances for the direct, intersection and Karnaugh methods. Figure 6(a,b) show that the direct and intersection methods have similar energy solutions for the ER and WS random trees. On the other hand, the Karnaugh method has larger energy solutions. The latter can be explained since the large number of used variables in the Karnaugh method, as can be seen in Fig. 4. We were unable to calculate the optimal energy solutions of the random instances since the large number of variables. The cases for *k* = 4, 5 were omitted since they have similar behavior.

The D-Wave SAPI also provides classical algorithms to solve QUBO/Ising problems by using simulated quantum annealing (SQA)^3^ on a Chimera graph of dimension 4 × 4 blocks. Figure 6(c,d) show the energy solutions obtained using D-Wave SAPI for ER and WS random trees, respectively. In this case, we use the same 80 random trees for *n* = 50 and *k* = 3 as in Fig. 6(a,b). For each instance of the MMC problem, 1000 readouts was requested to the SQA algorithm. Figure 6(c) shows the minimum energies among the requested 1000 readout per instance, for ER random trees. As can be seen in Fig. 6(c), the obtained energies have a similar behavior as in Fig. 6(a). Figure 6(a) also shows the energies for the case of WS random trees using the SQA algorithm. In this latter case, it was not possible to obtain the energies for the Karnaugh method since the embedding algorithm fails to embed large QUBO functions in a Chimera graph of a 4 × 4 dimension.

**Figure 7:**
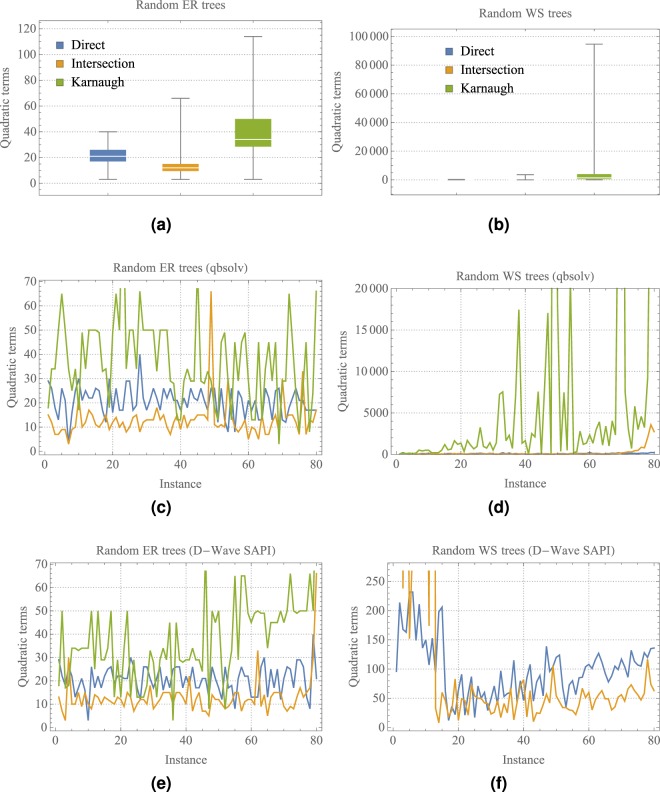
Visualisation of the number of quadratic terms based on the sorted energies shown in Fig. 6 over 80 random instances using the direct, intersection and Karnaugh QUBO constructions.

Figure 7 shows how the sorted energies in Fig. 6 are correlated with respect to the number of quadratic terms in the set of instances. Figure 7(a,b) present the distribution of quadratic terms for the direct, intersection and Karnaugh methods using random ER and WS trees in Fig. 6. Also, Fig. 7(c–f) show the number of quadratic terms of the direct, intersection and Karnaugh methods, where the instances were arranged based on the same energy sorting shown in Fig. 6(a–d), respectively. For instance, the higher energies in Fig. 6(a) are correlated with the higher number of quadratic terms generated by the Karnaugh method. The same correlation is observed for the classical solvers qbsolv and D-Wave simulator.

We have proposed QUBO formulations of the MMC problem restricted to the family of trees. Our simulation results show that the direct and intersection methods have a similar scaling, in the number of quadratic terms, and performance. On the other hand, the Karnaugh method has the worst results, mainly by the required large number of variables and quadratic terms in its construction.

## Discussion

We have proposed a method to formulate the Minimum Multicut Problem into the QUBO representation, and the technical difficulties faced when embedding and submitting a problem to the quantum annealer processor. We have considered a special NP − hard case of the Minimum Multicut problem based on random trees representations. This special case allows us to formulate a QUBO expression that can be embedded into the Chimera graph using a moderate number of qubits after the degree reduction of the high degree expression. Moreover, we have proposed the Karnaugh method to analytically construct instances of the MMC problem and the preliminary results of our algorithms are promising.
